# Recombinant Bri3 BRICHOS domain is a molecular chaperone with effect against amyloid formation and non-fibrillar protein aggregation

**DOI:** 10.1038/s41598-020-66718-y

**Published:** 2020-06-17

**Authors:** Helen Poska, Axel Leppert, Helene Tigro, Xueying Zhong, Margit Kaldmäe, Harriet E Nilsson, Hans Hebert, Gefei Chen, Jan Johansson

**Affiliations:** 1 School of Natural Sciences and Health, Tallinn University, Tallinn, Estonia; 2 Department of Neurobiology, Care Sciences and Society, Division of Neurogeriatrics, Karolinska Institutet, Huddinge, Sweden; 3 School of Engineering Sciences in Chemistry, Biotechnology and Health, Department of Biomedical Engineering and Health Systems, KTH Royal Institute of Technology, Department of Biosciences and Nutrition, Karolinska Institutet, Huddinge, Sweden; 4 Department of Microbiology, Tumor and Cell Biology, Karolinska Institutet Biomedicum, Solna, Sweden; 5 Present Address: Department of Neurobiology, Care Sciences and Society, Division of Neurogeriatrics, Karolinska Institutet, Huddinge, Sweden

**Keywords:** Biochemistry, Proteins, Chaperones

## Abstract

Molecular chaperones assist proteins in achieving a functional structure and prevent them from misfolding into aggregates, including disease-associated deposits. The BRICHOS domain from familial dementia associated protein Bri2 (or ITM2B) probably chaperones its specific proprotein region with high β-sheet propensity during biosynthesis. Recently, Bri2 BRICHOS activity was found to extend to other amyloidogenic, fibril forming peptides, in particular, Alzheimer’s disease associated amyloid-β peptide, as well as to amorphous aggregate forming proteins. However, the biological functions of the central nervous system specific homologue Bri3 BRICHOS are still to be elucidated. Here we give a detailed characterisation of the recombinant human (rh) Bri3 BRICHOS domain and compare its structural and functional properties with rh Bri2 BRICHOS. The results show that rh Bri3 BRICHOS forms more and larger oligomers, somewhat more efficiently prevents non-fibrillar protein aggregation, and less efficiently reduces Aβ42 fibril formation compared to rh Bri2 BRICHOS. This suggests that Bri2 and Bri3 BRICHOS have overlapping molecular mechanisms and that their apparently different tissue expression and processing may result in different physiological functions.

## Introduction

To maintain cellular homeostasis, organisms have evolved ways to prevent misfolded and aggregated proteins from causing cellular damage^1^. Molecular chaperones are part of this defence arsenal and can interfere with virtually all steps from co-translational protein folding to degradation, which promote the formation of correctly folded and functional proteins over-accumulation of self-assembled aggregates that may cause toxicity^1,2^.

Amyloid fibrils are the end-result of one type of misfolding and are composed of β-sheets in which the β-strands run perpendicular to the fibril axis^3^. Many proteins can form amyloid-like fibrils *in vitro*^4,5^ but tissue deposits of specific proteins in the amyloid state are coupled to only about 40 diseases, including many severe neurodegenerative disorders^6^. Alzheimer’s disease (AD) is the most common cause of dementia, and there is no disease-modifying treatment available. AD is histopathologically characterised by the presence of extracellular amyloid plaques mainly in the neocortex and hippocampus, and these plaques contain fibrils of the amyloid β-peptide 1–40 (Aβ40 and 1–42 (Aβ42)^7^. AD is also defined by the formation of another type of protein deposit—intracellular neurofibrillary tangles composed of hyperphosphorylated tau protein^8^. The formation of Aβ and tau deposits is thought to result in the loss of synapse function, eventually leading to cell death and brain atrophy. The exact mechanisms, by which protein misfolding and aggregation result in neuronal loss and dysfunction in AD are unresolved, but it is believed that the main toxic agents are soluble oligomeric forms of Aβ rather than the mature fibrils^9,10^.

The BRICHOS domain was initially defined by multiple amino acid sequence alignments of the proteins *Bri*2, *cho*ndromodulin-1 and pro*s*urfactant protein C (proSP-C)^11^. The BRICHOS domain has been found in 10 protein families in humans, and BRICHOS containing proproteins share a common architecture^12^. These proproteins contain specific regions with high β-sheet propensity, which BRICHOS is believed to chaperone during biosynthesis^13–15^. Mutations in the BRICHOS domain of proSP-C result in lethal disease already in infancy, associated with lung amyloid composed of the proSP-C β-prone region, which supports the hypothesis that BRICHOS is a chaperone that prevents amyloid toxicity^16^. Bri2 is expressed in the CNS and in peripheral tissues. Mutations that extend its β-prone region result in amyloid deposits and familial British and Danish dementia^17,18^. Recombinant human (rh) BRICHOS domains from Bri2 and proSP-C reduce amyloid fibril formation and neurotoxicity of Aβ40 and Aβ42^19–23^, and transgenic overexpression of BRICHOS reduces Aβ42 related pathology in fruit flies^21,24^. These findings suggest that BRICHOS domains are able to prevent fibril formation and toxicity of other clients than the β-prone regions of their respective proprotein. In support of this, rh proSP-C BRICHOS prevents fibril formation of medin, a fragment of lactadherin that forms amyloid in the human aortic wall^25^, and we have recently found that Bri2 BRICHOS potently inhibits amyloid formation and toxicity of type 2 diabetes associated islet amyloid polypeptide (IAPP)^26^.

The conservation of amino acid sequences between BRICHOS domains from different proproteins is low, but their predicted secondary structures are similar^12,14^, and three residues are strictly conserved - two Cys and one Asp residue^11,12^.

Bri3 is a 267-residue protein, and as other BRICHOS proteins, it contains an N-terminal part, a transmembrane segment, a linker region, the BRICHOS domain and a C-terminal segment. Bri3 is structurally similar to Bri2 with an overall 44% sequence identity^12,27^. However, Bri3 differs from Bri2 in several ways — it is exclusively expressed in the CNS, the C-terminal segment is cleaved by furin but the BRICHOS domain is apparently not released^28^, and it interacts with amyloid plaques and Aβ in different manners than Bri2^29^.

BRICHOS domains from different proproteins have been found to interfere with distinct steps in the reaction pathway from monomeric Aβ42 to fibrils. ProSP-C BRICHOS exclusively blocks the secondary nucleation step^23,30^. Bri2 BRICHOS, however, has shown a more complex inhibition effect by blocking both a surface catalysed secondary nucleation and elongation of fibrils^21,30^.

Moreover, Bri2 BRICHOS, but not the proSP-C BRICHOS domain also prevents non-fibrillar aggregation of partly denatured proteins^21^. These observations raise the question of how different types of chaperone activities of BRICHOS are mediated. Isolated rh Bri2 BRICHOS monomers potently prevent hippocampal neuronal network toxicity of Aβ42, while dimers strongly suppress Aβ42 fibril formation. The dimers assemble into high-molecular-weight oligomers with an apparent two-fold symmetry, which are efficient inhibitors of non-fibrillar protein aggregation. Thus Bri2 BRICHOS affects qualitatively different aspects of protein misfolding and toxicity via different quaternary structures^31,32^. Rh Bri3 BRICHOS also reduces Aβ42 fibril formation but less efficiently than Bri2 BRICHOS^29^ but the detailed effects of Bri3 BRICHOS on Aβ42 fibril formation have not been studied. It is not known whether it similarly to Bri2 BRICHOS also prevents non-fibrillar protein aggregation, or whether it like proSP-C BRICHOS exclusively affects fibril formation.

In this study, we sought a more comprehensive understanding of Bri3 BRICHOS and its ability to reduce Aβ42 amyloid fibril formation and non-fibrillar aggregation.

## Results and Discussion

### Homology model of Bri3 BRICHOS and comparison to Bri2 BRICHOS

**Figure 1:**
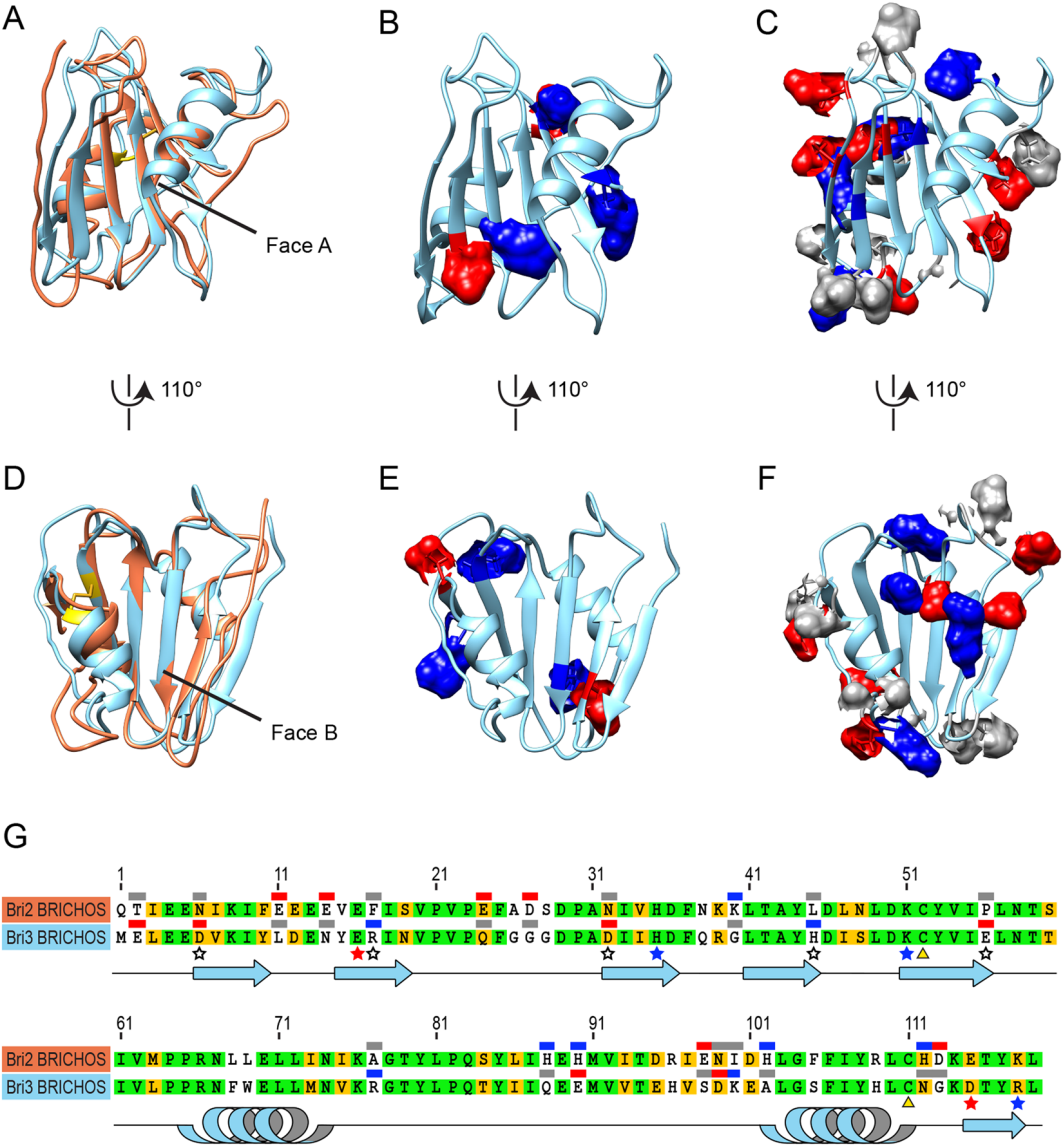
Structural models and sequence alignment of Bri2 and Bri3 BRICHOS domains. Modelled structures of Bri2 (orange) and Bri3 (light blue) BRICHOS domains are shown from face A **(a–c)** or face B **(d–f)**, and panels **a** and **d** show overlays of the two domains. The disulfide bond between the two strictly conserved cysteine residues (distance between the sulfur atoms of the two Cys is 2 Å) is highlighted in gold for Bri3 BRICHOS Residue side-chains that are highlighted by space-filling show either conserved **(b** and **e)** or changed (**c** and **f**, the residues shown are those present in Bri3 BRICHOS) charged residues between Bri2 and Bri3 BRICHOS, dark blue: positive charge, red: negative charge, grey: loss of charge to a neutral side-chain. **(g)** Sequence alignment of Bri2 and Bri3 BRICHOS. Shading represents identical residues (green) and similar residues (yellow), based on a Blosum62 score matrix with a threshold of 1. Residues indicated by small boxes above the sequences highlight the changed residues in panels **c** and **f** using the same colour-coding. A filled star highlights positions with conserved charge (also shown in **b** and **e**) and an open star indicates positions with varied charge (also shown in **c** and **f**) in the central β-sheet. The two conserved Cys are marked with yellow triangles.

Prediction using the I-TASSER web server^33^ gave similar overall tertiary structures for Bri3 and Bri2 BRICHOS (Fig. 1) with confidence scores (C-scores) of −1.37 for Bri2 BRICHOS and −1.42 for Bri3 BRICHOS. The only determined structure of a BRICHOS domain, from proSP-C, showed a central five-stranded β-sheet structure with one α-helix on each side^34^. Though the sequence identity between different BRICHOS domains is variable, for example between proSP-C and Bri2 BRICHOS it is less than 25%^20^, the secondary structure elements and tertiary structures are predicted to be conserved^12^. Many hydrophobic residues of β-sheet face A (see Fig. 1) of pro-SP-C BRICHOS are changed to charged residues in Bri2 BRICHOS, which correlates well with the physico-chemical properties of their supposed physiological target peptides (the hydrophobic transmembrane segment in case of proSP-C and the highly charged C-terminal Bri23 region in the case of Bri2), suggesting that face A is important for the substrate specificity^20^.

Sequence alignment of Bri2 and Bri3 BRICHOS (Fig. 1) shows that they share 58% identical residues and reveals that there are several residue replacements between the two domains where the physicochemical properties are largely unchanged, making 79% of residues in the two BRICHOS domains similar or identical. In total, 20 out of 51 non-identical residues include charge variations. Judging from the predicted structures most of the residue substitutions that result in different physicochemical properties in Bri3 BRICHOS compared to Bri2 BRICHOS are located in unstructured loops or in β-sheet with residue side chains orientated towards face B (Fig. 1c,f). More specifically, five residues with charge variations are in the central β-sheet and 15 are located in the loop regions, except for one residue that is in the helix 2 on the face B side. Side chains are clearly facing towards face B for 11 charge-changed residues, five of them are in β-sheet and six are around helix 2. From the x-ray structure of proSP-C BRICHOS^34^, it was suggested that face A of the β-sheet interacts with the client peptide regions during biosynthesis^14,20,34^. Face A harbours none of the now predicted non-conservative replacements between Bri2 and Bri3 BRICHOS but instead has four out of five conserved charged residues in the central β-sheet (Fig. 1b,c,e). The 23-residue proposed client peptide regions of Bri2 and Bri3 contain 18 identical residues. These observations suggest that Bri2 and Bri3 BRICHOS domains may have similar functions in terms of interacting with their respective β-prone region during proprotein biosynthesis, and that they have diverged during evolution primarily by varying the loop regions and the face B of the β-sheet. Further experiments, in particular changing the physicochemical properties of either side of the central β-sheet in different BRICHOS domains by site-directed mutagenesis will be needed to test this hypothesis.

### Characterisation of recombinant human (rh) Bri3 BRICHOS domain

**Figure 2:**
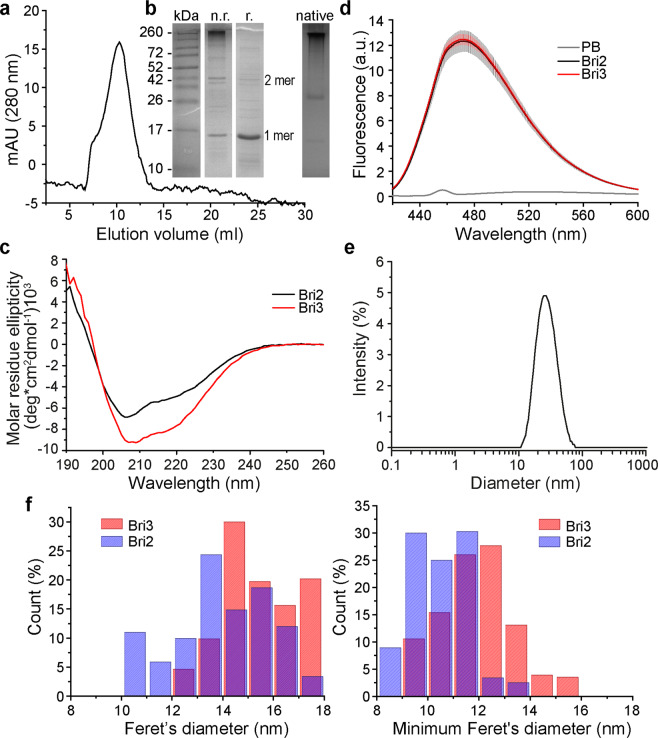
Characterisation of rh Bri3 BRICHOS and comparison to rh Bri2 BRICHOS. **(a)** SEC using a Superose 6 column, **(b)** non-reducing (n.r.) and reducing (r.) SDS-PAGE and native PAGE of rh Bri3 BRICHOS high molecular weight oligomer fraction from SEC. Full size gels are presented in Supplementary Fig. S8a. **(c)** CD spectra of rh Bri3 BRICHOS (red) and rh Bri2 BRICHOS (black) measured at 25 °C. **(d)** Fluorescence emission of 2 μM bis-ANS in phosphate buffer (PB, grey) and in the presence of 1 μM rh Bri3 BRICHOS (red) or rh Bri2 BRICHOS (black). **(e**) DLS lognormal size intensity distribution of 10 µM rh Bri3 BRICHOS. **(f)** Histograms with 1 nm binning interval of Feret’s and minimal Feret’s diameters of rh Bri2 or Bri3 BRICHOS based on 2D classification.

Size exclusion chromatography (SEC) (Fig. 2a, Supplementary Fig. S1) and native PAGE (Fig. 2b) show that rh Bri3 BRICHOS migrates mainly as high molecular weight oligomers. SDS-PAGE under non-reducing conditions shows that disulfide bonds to a significant extent hold together the larger aggregates. Also, monomers, dimers and smaller oligomers are detected by SDS-PAGE under non-reducing conditions (Fig. 2b), which indicates that non-covalent inter-subunit interactions contribute to the formation of the high molecular weight oligomers. Rh Bri3 BRICHOS monomer has a calculated mass of 17.3 kDa, and it migrates as expected on SDS-PAGE (Fig. 2b). Comparison of the SEC elution volume to standards with known hydrodynamic Stokes radii gives an apparent diameter of 17 nm for the Bri3 oligomers. This result is in a good agreement with diameters obtained from transmission electron micrographs (average maximum diameter ± SD: 15 ± 1 nm) and from dynamic light scattering data (26 ± 11 nm, polydispersity index 0.163) (Fig. 2e, Supplementary Fig. S1c). Rh Bri2 BRICHOS has an oligomer diameter of 14 ± 2 nm. Comparison of minimum and maximum Feret’s diameters, indicates that rh Bri3 BRICHOS forms larger oligomers compared to rh Bri2 BRICHOS (Fig. 2f). The same tendency is also observed when comparing rh Bri3 BRICHOS and rh Bri2 BRICHOS by SEC; rh Bri3 BRICHOS shows a slight shift towards larger species and relatively more of it elutes in the void volume compared to rh Bri2 BRICHOS (Supplementary Fig. S1b).

Circular dichroism (CD) spectroscopy of rh Bri3 BRICHOS shows that its overall secondary structure content is similar to that of rh Bri2 BRICHOS (Fig. 2c) and to isolated rh Bri2 BRICHOS oligomers^19,31^, but with somewhat increased residual molar ellipticity in the 205–225 nm regions. The CD spectra suggest that rh Bri3 BRICHOS is folded into a structure with a mixture of α-helices and β-sheet, which is compatible with the model structure (Fig. 1).

To probe for the presence of exposed hydrophobic surfaces, the hydrophobic fluorescent dye bis-ANS^35^ was used. Incubation of rh Bri3 BRICHOS with bis-ANS shows a considerable increase in emission intensity and a marked blue shift in emission maximum to 472 nm, compared to bis-ANS in buffer; very similar to the behaviour of rh Bri2 BRICHOS (Fig. 2d). Bis-ANS binding to proteins is dominated by hydrophobic interactions, and the spectral changes thus indicate that rh Bri3 BRICHOS exposes hydrophobic regions.

### Effect of rh Bri3 BRICHOS on aggregation of partly denatured proteins

**Figure 3:**
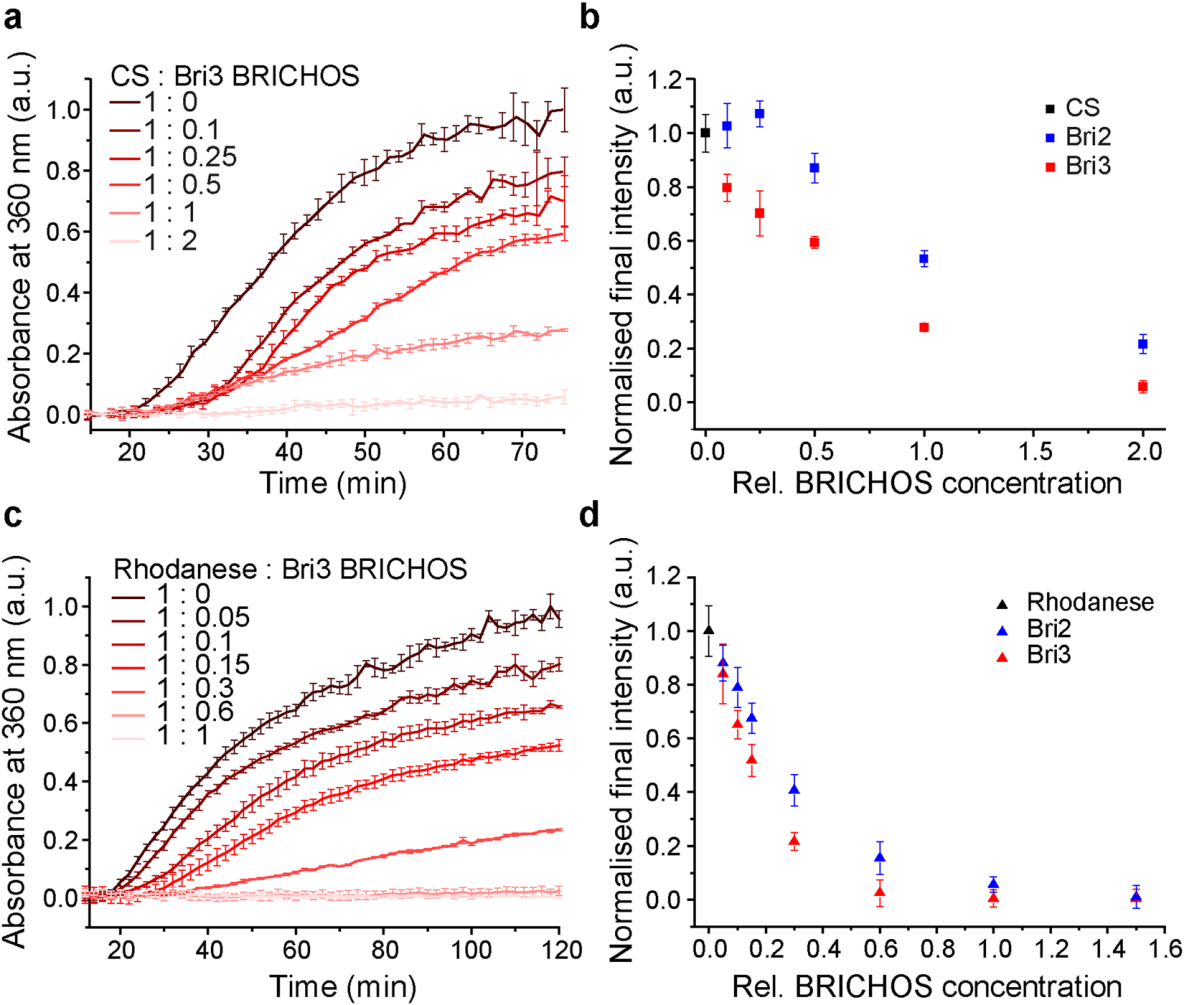
Rh Bri3 BRICHOS suppresses thermally induced non-fibrillar aggregation of model substrates. (**a**) Aggregation of 600 nM citrate synthase (CS) alone (dark brown) and in the presence of different molar ratios of rh Bri3 BRICHOS (shades of red) at 45 °C. Representative data for triplicates shown as mean ± SD. (**b)** The final intensity of CS aggregation alone (black) or in the presence of different molar ratios of rh Bri3 (red) or Bri2 BRICHOS (blue). **(c)** Aggregation of 3 µM rhodanese alone (dark brown) or in the presence of different molar ratios of rh Bri3 BRICHOS (shades of red) at 45 °C presented as mean ± SD of triplicates. **(d)** The final intensity of rhodanese aggregation alone (black) or in the presence of different molar ratios of rh Bri3 (red) and Bri2 BRICHOS (blue). The data in panels (**b**) and (**d**) are baseline corrected and normalised to the mean of CS or rhodanese maximum intensity, respectively, and presented as mean ± SD of 3 experiments performed in triplicates.

BRICHOS domains from different proproteins have different chaperone activities; rh proSP-C BRICHOS for example, is essentially unable to prevent non-fibrillar protein aggregation while rh Bri2 BRICHOS efficiently suppresses aggregation of partly denatured proteins including citrate synthase (CS)^21^. For Bri2 BRICHOS, the activity against nonfibrillar protein aggregation is almost entirely mediated by oligomers while dimers and monomers are essentially inactive^31^. Rh Bri3 BRICHOS efficiently suppress aggregation of partly heat-denatured CS in a concentration-dependent manner, with clear effects already at sub-stoichiometric ratios (Fig. 3a). Rh Bri3 BRICHOS mostly forms high molecular weight disulphide-linked oligomers (Fig. 2, Supplementary Fig. S1) and no difference in chaperone activity is seen between the unresolved crude form of Bri3 BRICHOS and the high molecular weight fractions isolated by SEC (data not shown). Bri3 BRICHOS is able to almost completely prevent the aggregation of CS at 1:1 molar ratio, while for Bri2 BRICHOS two-fold molar excess is needed for the same effect (Fig. 3b, Supplementary Fig. S2a). Likewise, rh Bri3 BRICHOS prevents aggregation of thermo-destabilized rhodanese already at substoichiometric amounts and is somewhat more efficient against non-fibrillar aggregation of also this model protein (Fig. 3c,d, Supplementary Fig. S2b). Rh BRICHOS concentrations were also measured with BCA and Bradford assays and the results were compared to spectrophotometric concentration determinations (Supplementary Fig. S3). There results show, that independent of method used for protein concentration determination, rh Bri3 BRICHOS is somewhat more effective than rh Bri2 BRICHOS in preventing thermal aggregation of CS (Supplementary Fig. S4).

**Figure 4:**
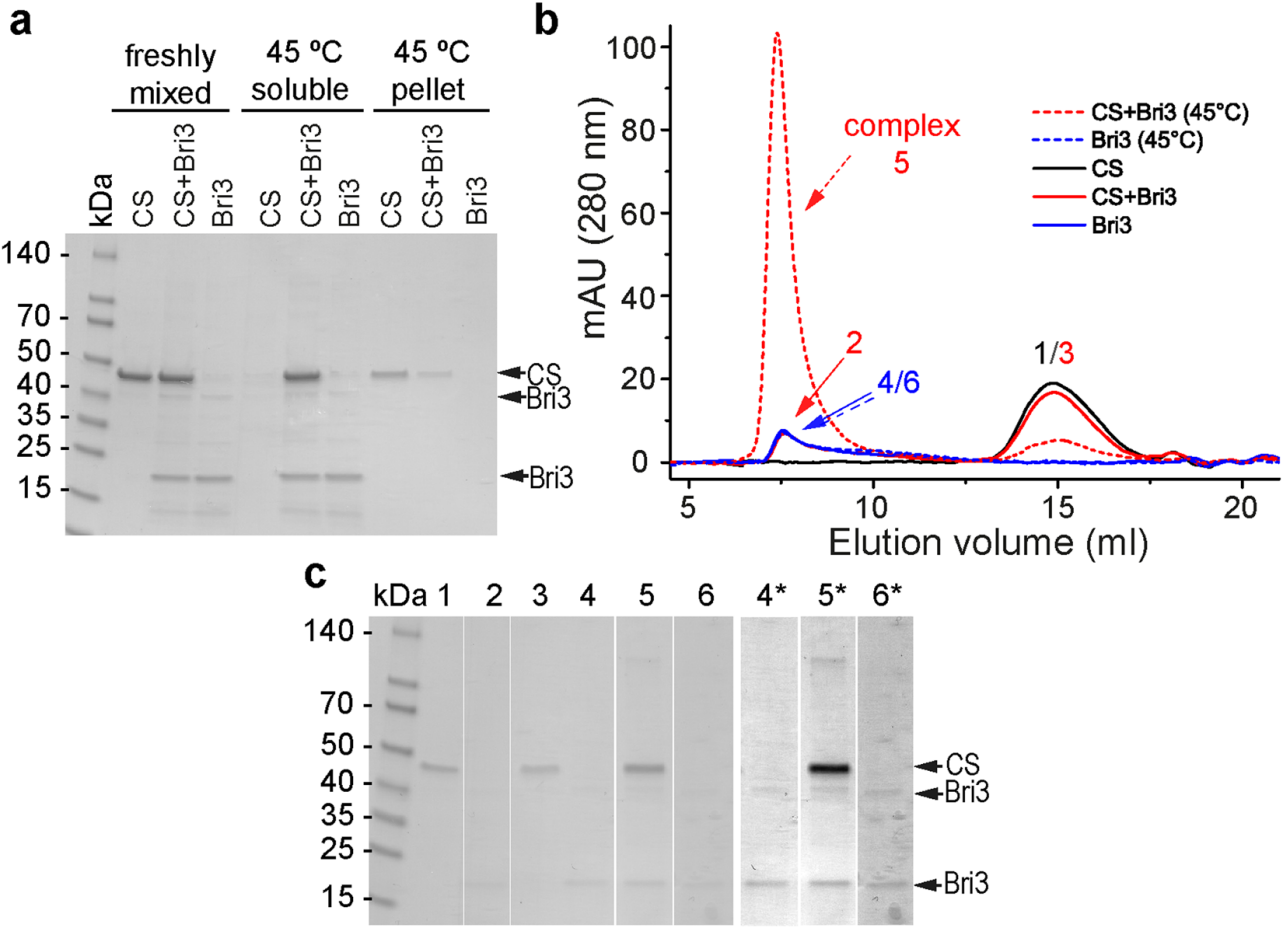
Rh Bri3 BRICHOS forms a complex with partly denatured CS. **(a)** Reducing SDS-PAGE of freshly prepared 1:2 molar ratio of CS:rh Bri3 BRICHOS, CS and rh Bri3 BRICHOS alone and soluble as well as pellet fractions after 2 h incubation at 45 °C. **(b)** SEC on Superose 6 column of 1200 nM CS alone (black line), of a freshly mixed 1:2 molar ratio of CS:rh Bri3 BRICHOS (solid red line), rh Bri3 BRICHOS alone (solid blue line), and of the supernatants after heat treatment of a 1:2 molar ratio mixture of CS:rh Bri3 BRICHOS (dashed red line), and of rh Bri3 BRICHOS alone (dashed blue line). **(c)** Numbered SEC peak fractions in (**b**) were analysed by SDS-PAGE under reducing conditions. Non-adjacent lanes from the gel are separated by white vertical spaces; the lanes marked with * have been manipulated to increase the contrast. Full size gels are presented in Supplementary Fig. S8b,c.

In order to study potential complex formation between rh Bri3 BRICHOS domain and CS after thermal aggregation, samples were collected after completion of aggregation assay of a CS:BRICHOS mixture at 1:2 molar ratio, spun down and the supernatant as well as pellet fractions were analysed by SDS-PAGE and the supernatant was analysed by SEC. The results show that rh Bri3 BRICHOS forms a complex with thermo-destabilized CS that elutes in the void volume (Fig. 4b), which can explain how rh Bri3 BRICHOS keeps CS in a soluble state. In contrast, after heat treatment of CS alone essentially all protein is in the pellet and no protein is found in the soluble fraction (Fig. 4a). Freshly mixed samples gave separate peaks for CS and rh Bri3 BRICHOS upon SEC and non-heat-treated CS elutes on SEC and migrates on SDS-PAGE as an about 45 kDa protein, compatible with a monomer (Fig. 4c). Most CS is detected in complex with rh Bri3 BRICHOS (Fig. 4), while as shown in the earlier study, only a fraction of CS co-migrated with rh Bri2 BRICHOS despite that a larger excess of CS was used^21^.

The rh Bri2 and Bri3 BRICHOS domains seem to preserve their folded states with only minor changes occurring at the elevated temperatures used in the aggregation assays, as judged from their CD spectra and native-PAGE (Supplementary Fig. S5). Overall, rh Bri3 BRICHOS is an efficient molecular chaperone that is able to suppress thermally induced amorphous aggregation of different substrates and can form a stable complex with thermally destabilized CS.

### Effects of rh Bri3 BRICHOS on kinetics of Aβ42 fibril formation

Amyloid fibril formation can be suppressed by molecular chaperones via different mechanisms. Different BRICHOS domains influence distinct steps in the fibrillization pathway from monomeric Aβ42 to amyloid fibrils^21,30^. ProSP-C BRICHOS for example exclusively inhibits the secondary nucleation, thereby blocking the main source of toxic Aβ42 oligomers^30^, while Bri2 BRICHOS blocks both the secondary nucleation and the elongation of fibrils^21,30^.

**Figure 5:**
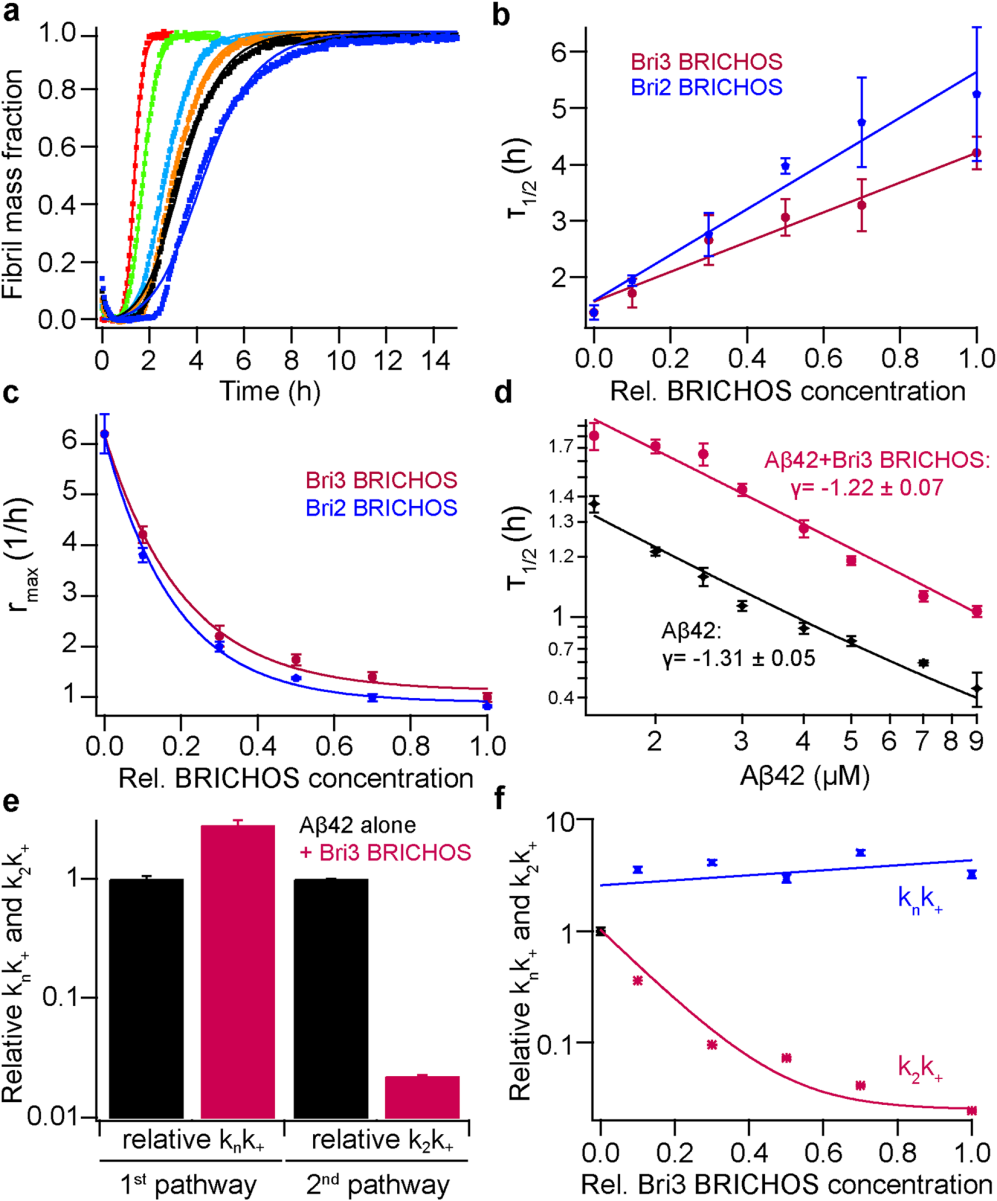
Rh Bri3 BRICHOS inhibits Aβ42 fibril formation by affecting secondary pathways. (**a**) Individual fits (solid lines) of normalized and averaged aggregation traces (dots) of 3 µM Aβ42 in the presence of 1:0 (red), 1:0.1 (green), 1:0.3 (cyan), 1:0.5 (orange), 1:0.7 (black) and 1:1 (blue) molar ratios of rh Bri3 BRICHOS with the combined rate constants $\sqrt{{k}_{n}{k}_{+}}$ and $\sqrt{{k}_{+}{k}_{2}}$ as free fitting parameters. Values for **(b)** *τ*_*1/*2_ and **(c)** r_max_ were extracted from the sigmoidal fitting of Aβ42 aggregation traces in the presence of rh Bri3 BRICHOS (red) (data shown in panel **a**) or in the presence of rh Bri2 BRICHOS (blue) (data shown in Supplementary Fig. S6). **(d)** Aβ42 in the absence (black) and presence (red) of 3 µM rh Bri3 BRICHOS exhibits a similar dependence of the aggregation half time described by the γ-exponent. **(e)** Global analysis from the data sets in Fig. 6a,b revealed a dominant effect rh Bri3 BRICHOS on $\sqrt{{k}_{+}{k}_{2}}$, related to secondary nucleation. **(f)** The dependencies of the relative combined rate constants obtained from the fits of rh Bri3 BRICHOS in panel (**a**) reveal a strong effect of rh Bri3 BRICHOS on secondary nucleation (*k*_*2*_*k*_+_) but not on primary (*k*_*n*_*k*_+_) pathways.

To find out how rh Bri3 BRICHOS affects Aβ42 fibrillization *in vitro*, we first incubated 3 µM Aβ42 in the absence and in the presence of rh Bri3 BRICHOS from 1:0.1 to 1:1 Aβ42:BRICHOS molar ratios and the formation of fibrils was monitored by thioflavin T (ThT) fluorescence. Rh Bri3 BRICHOS shows a dose-dependent reduction of Aβ42 fibril formation already at substoichiometric concentrations (Fig. 5a) as also seen for rh Bri2 BRICHOS (Fig. 5, Supplementary Fig. S6). The fibrillization half time, τ_1/2_, was increased linearly against the concentration of rh Bri3 BRICHOS (Fig. 5b), while the maximum rate of aggregation, r_max_, shows a mono-exponential dependence (Fig. 5c). The effects of rh Bri3 BRICHOS on τ_1/2_ are smaller than what is seen for the rh Bri2 BRICHOS (Fig. 5b); both are smaller compared to an unresolved mixture of rh Bri2 BRICHOS assembly states^21^, and they are also smaller than the effects of isolated rh Bri2 BRICHOS oligomers on Aβ42 τ_1/2_^31^. The γ-value, which describes the aggregation mechanism of Aβ42^36^, is similar in the absence and presence of rh Bri3 BRICHOS (Fig. 5d), suggesting that aggregation follows mainly secondary pathways in both cases. Electron microscopy shows that the morphology of Aβ42 fibrils formed in the absence or presence of rh Bri3 BRICHOS (Supplementary Fig. S7) is similar.

**Figure 6:**
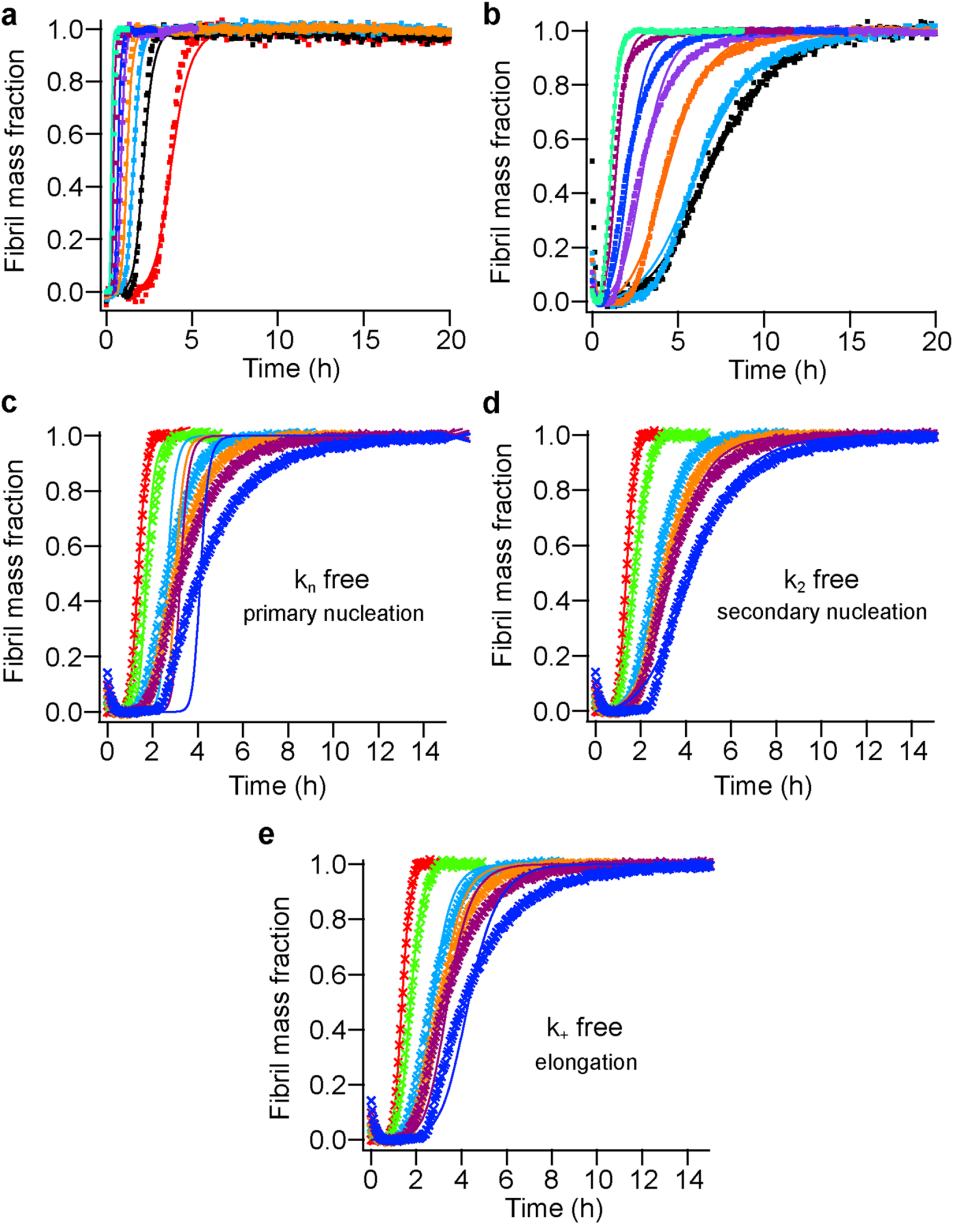
Aggregation kinetics of Aβ42 in the presence and absence of rh Bri3 BRICHOS. (**a-b**) Global fits (solid lines) of aggregation traces (dots) with different Aβ42 concentrations from 1.5 (red), 2.0 (black), 2.5 (cyan), 3.0 (orange), 4.0 (purple), 5.0 (blue), 7.0 (dark red) to 9 (green) µM in the absence (**a**) or in the presence (**b**) of 3 µM rh Bri3 BRICHOS, where $\sqrt{{k}_{n}{k}_{+}}$ and $\sqrt{{k}_{+}{k}_{2}}$ are constrained to the same value across all concentrations. (**a**) Aβ42 alone, $\,\sqrt{{k}_{n}{k}_{+}}$ = 6.4 ±0.16  M^−1^ S^−1^, $\sqrt{{k}_{+}{k}_{2}}$ = 2.74 × 10^5^ ± 8.5 M^−3/2^ S^−1^, *χ*^2^ =4.1. (**b**) Aβ42 with rh Bri3 BRICHOS, $\,\sqrt{{k}_{n}{k}_{+}}$ = 10.8 ± 0.53 M^−1^ S^−1^, $\sqrt{{k}_{+}{k}_{2}}$ = 0.4 × 10^5^ ± 413 M^−3/2^ S^−1^, *χ*^2^ =2.3. **(c-e)** Aggregation kinetics of 3 µM Aβ42 in the presence of different molar ratios of rh Bri3 BRICHOS (Aβ42:rh Bri3 BRICHOS): 1:0 (red), 1:0.1 (green), 1:0.3 (cyan), 1:0.5 (orange), 1:0.7 (plum) and 1:1 (blue). The global fits (solid lines) of the aggregation traces (crosses) were constrained such that only one single rate constant is the free fitting parameter. *χ*^2^ values describing the quality of the fits are 13.69 for *k*_*n*_ free, 0.68 for *k*_2_ free and 2.79 for *k*_+_ free, respectively.

To identify microscopic processes in the Aβ42 fibrillization, we performed kinetic experiments at different Aβ42 concentrations with 3 µM rh Bri3 BRICHOS and fitted the results with a nucleation model, where $\sqrt{{k}_{n}{k}_{+}}$ (for primary pathways) and $\sqrt{{k}_{+}{k}_{2}}$ (for secondary pathway) are constrained globally (Fig. 6a,b). The kinetic fitting showed that the parameter $\sqrt{{k}_{n}{k}_{+}}$ is not much affected, whereas $\sqrt{{k}_{+}{k}_{2}}$ is markedly reduced by rh Bri3 BRICHOS (Fig. 5e). We also performed the kinetic analysis with the dataset for constant Aβ42 concentration and different rh Bri3 BRICHOS concentrations, which allows to elucidate the quantitative effects, by leaving both $\sqrt{{k}_{n}{k}_{+}}$ and $\sqrt{{k}_{+}{k}_{2}}$ free. This likewise showed that the $\sqrt{{k}_{2}{k}_{+}}$ was changed significantly while $\sqrt{{k}_{n}{k}_{+}}$ did not (Fig. 5f), suggesting again that rh Bri3 BRICHOS mainly affects the secondary pathway during Aβ42 fibrillization.

To study how the individual microscopic rate constants (*k*_*n*_, *k*_+_ or *k*_2_) for Aβ42 fibrillization are affected by rh Bri3 BRICHOS, global kinetic analysis was performed on the dataset for constant Aβ42 concentration and different rh Bri3 BRICHOS concentrations, leaving only one fitting rate, *k*_2_*, k*_+_, or *k*_*n*_, free (Fig. 6c–e). Both *k*_2_ and *k*_+_ but not *k*_*n*_ describe well the time evolution of Aβ42 fibril formation in the presence of rh Bri3 BRICHOS, with 𝜒^2^ of 0.68, 2.79 and 13.69, respectively. This suggests that rh Bri3 BRICHOS mainly inhibits secondary nucleation as well as the elongation process during Aβ42 fibrillization, which is similar as for rh Bri2 BRICHOS^21,30^.

### Functions of Bri3 and Bri2 and their BRICHOS domains

Bri2 and its BRICHOS domain have been proposed to play crucial roles in various aspects of Alzheimer disease^37^. Bri3 is homologous to Bri2 with 44% overall sequence identities^27^, which suggest that the two proteins have similar functions. Both Bri2 and Bri3 are transported through the secretory pathway and are present in the plasma membrane, but they appear to be processed partly different^28^. Bri2 goes through a C-terminal cleavage by furin or furin-like convertases in the Golgi apparatus, resulting in a small secreted peptide and mature membrane-bound Bri2^38^, which then undergoes shedding of its ectodomain by ADAM10^39^, thereby releasing a soluble extracellular Bri2 BRICHOS domain. Bri3 is cleaved by furin^38^ but fails to undergo shedding of the BRICHOS domain in transfected HEK293 cells^28^. It appears form the present study that the Bri2 and Bri3 BRICHOS domains are quite similar in structures and functions, while the entire Bri2 and Bri3 proteins, and particularly their expression and processing seem more likely to generate potentially different physiological functions.

Both Bri2 and Bri3 interact with the Aβ precursor protein (APP) and regulate its processing and thereby have an effect on Aβ production^40–43^. Furthermore, it has been shown that the furin cleaved mature Bri2 is localised in the plasma membrane and in endocytic vesicles and there interacts with APP^41,43^. Bri3 also binds to APP^40^ and similar to Bri2, interferes with APP processing by specifically binding to mature APP rather than having a direct effect on secretase activities. For both Bri3^42^ and Bri2^44^, the region that binds APP is located in the juxtamembrane linker region and APP binding is thus not directly dependent on the presence of the BRICHOS domain.

A recent study demonstrated that different assembly states of rh Bri2 BRICHOS domain have distinct activities: monomers most efficiently inhibit Aβ42 neurotoxicity, dimers are best in reducing fibril formation, and oligomers are by far most efficient against nonfibrillar aggregation^31^. The present study shows that also rh Bri3 BRICHOS has potent molecular chaperone activities. Bri3 BRICHOS forms mainly high molecular weight disulphide linked assemblies and only minor amounts of smaller species like dimers and monomers are observed (Fig. 2). The observed differences between rh Bri3 and Bri2 BRICHOS chaperone profiles shown here (Figs. 3 and 5) and references^21,31^ can thus be rationalized by the higher tendency of Bri3 BRICHOS to form larger assemblies compared to Bri2 BRICHOS (Fig. 2f, Supplementary Fig. S1). For rh proSP-C BRICHOS, it has been shown that trimer is a relatively inactive storage form while the monomer is more efficient in delaying Aβ42 aggregation^45^. The lower potency of rh Bri3 BRICHOS against Aβ42 fibril formation can be rationalized along the same line, *i.e*. that rh Bri3 BRICHOS forms less monomers and dimers than rh Bri2 BRICHOS.

The two BRICHOS domains share about 60% identical residues. The face A of Bri2 and Bri3 BRICHOS is well conserved, it harbours no residue exchanges that result in significant changes in physicochemical properties, while for the face B there are abundant residue replacements that change the properties of the side-chains (Fig. 1). The overall net charge of face B however is essentially unchanged. We speculate that the divergent charge distribution of face B of Bri2 versus Bri3 BRICHOS might influence their different tendencies to form larger oligomeric species, which in turn affects activity against non-fibrillar as well as fibrillar protein aggregation. Our present data suggests that rh Bri3 BRICHOS distribution towards larger oligomers makes it a more efficient chaperone against non-fibrillar aggregation. However, additional factors, like e.g. hydrophobicity of exposed residues and their distribution in the Bri2 and Bri3 structures likely also influence their capacities as molecular chaperones. Further studies, e.g. using site- and motif-specific mutants are warranted to understand the mechanisms that underlie the regulation of assembly states and how they affect BRICHOS chaperone activity.

## Material and Methods

### Bioinformatic analysis

Tertiary structure predictions of Bri2 BRICHOS (113–231) and Bri3 BRICHOS (112–230) were performed using the I-TASSER web server^33^. Structures with the highest C-score were visualized and aligned using the software UCSF Chimera^46^. The confidence scores (C-score) of the predicted models were: −1.37 for Bri2 BRICHOS and −1.42 for Bri3 BRICHOS. The I-Tasser database does not predict the formation of disulphide bonds. Therefore, we measured the distance between the sulphur groups of the two only cysteine residues and assumed that a disulphide bond is formed when the distance is below 2.3 Å. The measured distance between the sulphur groups of the Bri2 BRICHOS model was 2.019 Å, and that for the Bri3 BRICHOS model was 2.016 Å.

The amino acid sequences of Bri2 and Bri3 BRICHOS have been aligned with Geneious R7.1 using a Blosum62 score matrix.

### Expression and purification of recombinant BRICHOS proteins

Gene sequences coding for the human, Bri3(112-230) and Bri2(90–236) were ligated to pET-32c(+) vector coding for thioredoxin (Trx), hexahistidine (His6), and S tags upstream of the cDNA insertion site. The Trx-His-S-tag-BRICHOS fusion proteins were expressed in *Escherichia coli* Shuffle T7 cells; thrombin was used to release Trx-His- from S-tag-BRICHOS. Bacteria were cultured in Luria-Bertani (LB) medium with 100 μg/ml ampicillin at 30 °C, overnight (o/n) with constant stirring. Expression was induced at an OD_600_ = 0,6 − 0,8 by adding isopropylthiogalactoside (IPTG) to 0.4 mM and the bacteria were grown for another 4 h. Cells were harvested by centrifugation at 6000 × g for 15 min at 4 °C; pellets were suspended in 0.15 M NaCl, 20 mM sodium phosphate buffer (PB), pH 7.4 and stored at −20 °C.

Purification of rh Bri3 and Bri2 BRICHOS was performed essentially as described by Peng *et al*.^19^ for Bri2 BRICHOS. Briefly, cells were lysed by sonication with Sonopuls HD2070 homogeniser (Bandelin electronic, Germany) for 3 min (1 sec on, 1 sec off for 1 min, then left to stand for 30 sec and repeated twice) and centrifuged at 6000 × g for 20 min at 4 °C. Pellets were suspended in 2 M urea in 0.15 M NaCl, 20 mM PB, pH 7.4, and sonicated as described above for 5 min and centrifuged at 15557 × g for 30 min at 4 °C. The collected supernatant was filtered through a 5 μm filter and frozen at −20 °C o/n. Next day, the thawed supernatant was poured onto an IMAC Ni-Sepharose 6 Fast Flow column (GE Healthcare, UK; Qiagen, Germany) pre-equilibrated with 2 M urea in 0.15 M NaCl, 20 mM PB, pH 7.4 and washed 3 times in series with 50 ml 0.15 M NaCl, 20 mM PB containing either 2 M and 1 M urea or 50 mM imidazole in final wash. The target fusion protein was then eluted with 300 mM imidazole 0.15 M NaCl, 20 mM PB, pH 7.4 and dialyzed against 20 mM PB, pH 7.4, while cleaving with thrombin (enzyme/substrate weight ratio of 0.002) for 16 h at 4 °C to remove the Trx and His6 tag. Molar extinction coefficients at 280 nm for measuring concentrations of for Bri3 and Bri2 fusion proteins were 31400 M^−1^ cm^−1^ and 25900 M^−1^cm^−1^ respectively.

After dialysis, the protein solution was reapplied to the Ni-Sepharose column and rh (S-tag) Bri3 BRICHOS were eluted with 20 mM PB, pH 7.4 and stored at −20 °C. Before the experiments, buffer exchange to 20 mM PB, 0.2 mM EDTA, pH 8.0 was carried out using PD-10 columns (GE Healthcare Life Sciences). Protein concentration at 280 nm was determined with a UV/Vis nanophotometer using a molar extinction coefficient 17545 M^−1^cm^−1^ for rh Bri3 and 12045 M^−1^cm^−1^ for rh Bri2 BRICHOS. Final yields of rh Bri3 and Bri2 BRICHOS were 3–5 mg from 500 ml of bacterial culture, and the freshly purified samples were frozen at concentrations of 50–70 μM.

### Expression and purification of recombinant Aβ peptide 1–42

Rh Met-Aβ1-42 (hereafter Aβ42) was expressed in *E. coli* BL21 using ion exchange and size exclusion steps as described before^47^. For Aβ42 monomer isolation, crude proteins were lyophilized and then dissolved in 7 M GuHCl and run through Superdex 75 10/300 GL column (GE Healthcare Life Sciences, UK) with 20 mM PB pH 8.0, 0.2 mM EDTA, 0.02% NaN_3_. The peptide concentration was determined by using an extinction coefficient of 1424 M^−1^cm^−1^ at A280-A300 nm. Purified monomers were aliquoted in protein low-binding tubes.

### PAGE on BRICHOS proteins

For sodium dodecyl sulphate polyacrylamide gel electrophoresis (SDS-PAGE) under reducing conditions, SDS loading buffer together with dithiothreitol (DTT) was added to samples and heated at 95 °C for 5 min. For non-reducing SDS-PAGE, samples were mixed with SDS loading buffer lacking DTT and treated as above. Samples were separated on 4–20% gradient gels, visualised with Coomassie blue staining, and documented. For the native PAGE samples were mixed with native loading buffer, without DTT and SDS and separated on 10% gel.

### Circular Dichroism spectroscopy

Circular Dichroism (CD) spectra for 10 µM Bri3 BRICHOS in 20 mM PB, 0.2 mM EDTA, pH 8.0 were recorded at 25 °C using Chirascan spectrometer (Applied Photophysics). Absorbance spectra from 260 to 180 nm with a wavelength increment of 1 nm were recorded in 1 mm path length quartz cuvettes. The bandwidth was set to 1 nm. Spectra shown are averages of 5 consecutive scans. As controls, rh Bri3 and rh Bri2 BRICHOS CD spectra were also recorded at 25 °C, after 24 h of incubation at 37 °C and after 2 h at 45 °C using a J-1500 CD spectrometer (JASCO) with a PTC-517 Peltier thermostat cell holder. CD spectra were recorded in 1 mm path length quartz cuvettes using wavelength from 260 to 175 nm with an increment of 0.5 nm. The bandwidth was set to 1 nm.

### Bis-ANS fluorescence

Bis-ANS fluorescence emission spectra were recorded in quartz glass cuvettes using a Fluorolog- 3 spectrofluorometer (FL3-22, Horiba Jobin Yvon). Fluorescence was excited at 395 nm and emission spectra recorded from 420 nm to 600 nm. The sample temperature was adjusted to 25 °C using a Peltier sample cooler. 1 µM rh Bri3 BRICHOS or rh Bri2 BRICHOS together with 2 µM bis-ANS were prepared in 20 mM phosphate buffer, 0.2 mM EDTA, pH 8 and measured after preincubation for 10 min at 25 °C.

### Dynamic light scattering

Dynamic light scattering (DLS) experiments were performed on a Zetasizer µV instrument (Malvern Inc., Worcestershire, UK) at 25 °C using the built-in standard values of the refractive index (1.330), viscosity (0.8872 cP) and material refractive index (1.45) for water. Protein samples were diluted in 20 mM phosphate buffer, 0.2 mM EDTA, pH 8 to a final concentration of 10 µM (Bri3 BRICHOS). The data represents the average of 15 measurements. Each measurement consists of 10 consecutive runs recorded for 10 seconds each.

### Transmission electron microscopy of Bri2 and Bri3 BRICHOS oligomers and Aβ42 fibrils

Rh Bri3 BRICHOS and rh Bri2 BRICHOS oligomers were isolated in 20 mM ammonium acetate pH 8.0 with a Superose 6 10/300 GL column. A narrow middle fraction of the broad main peak was collected and immediately stored on ice; the protein concentration was around 15 − 30 ng/μL. Samples of Aβ42 fibrils in the absence and presence of rh Bri3 BRICHOS were prepared after incubation of 5 µM Aβ42 monomers without or with 70% molar ratio of BRICHOS in 160 µl for 18 h. The samples were centrifuged at 22000 × g for 1 h at 4 °C. The pellet was resuspended in 20 µl 1x Tris-buffered saline. After sample preparation, 2 μL aliquot was adsorbed onto glow-discharged continuous carbon-coated copper grids (400 mesh, Analytical Standards) for 1 min. The grids were subsequently blotted with a filter paper, washed with two drops of Milli-Q water, and negatively stained with one drop of uranyl acetate [2% (w/v)] for 45 sec before final blotting and air-drying. The samples were imaged using a JEOL JEM2100F field emission gun transmission electron microscope (JEOL, Japan) operating at 200 kV. Single micrographs of the sample were recorded on a Tietz 4k × 4k CCD camera, TVIPS (Tietz Video and Image Processing Systems, GmbH, Gauting, Germany) at magnification of 85,000 (pixel size 1.76) and 1.5–3 μm defocus was used and a total of 12 and 8 images were recorded for rh Bri2 and Bri3 BRICHOS, respectively.

All rh Bri2 and Bri3 BRICHOS oligomer images were imported to EMAN2 (version 2.3) for further processing. After importing and estimating defocus with e2evalimage.py single particles in different orientations were selected from the images using e2boxer.py in manual mode, resulting in 2385 particles for rh Bri2 BRICHOS and 2394 particles for rh Bri3 BRICHOS. For each image, the contrast transfer function (CTF) parameters were estimated on boxed out regions containing particles (288 × 288 pixels for Bri2 and 240 × 240 pixels for Bri3) using the e2ctf.auto.py program. A reference-free 2D classification was performed afterwards. The Feret’s and minimal Feret’s diameters of 30 obtained 2D oligomer classes for each rh Bri2 and Bri3 BRICHOS were measured by bordering the oligomers and manual thresholding using Fiji ImageJ (National Institutes of Health) software.

### Analysis of chaperone activity using citrate synthase and rhodanese

Thermal aggregation of citrate synthase (CS) from porcine heart (Sigma-Aldrich, Germany) was monitored by following the light scattering at 360 nm^48^. After buffer exchange to 40 mM HEPES/KOH pH 7.5 by PD-10 (GE Healthcare Life Sciences) chromatography CS was diluted to an end concentration of 600 nM. Rhodanese from bovine liver was purchased from Sigma (Merck; Darmstadt, Germany), dissolved in PBS, pH 7.4 and concentration was determined by measuring the absorbance at 280 nm and using an extinction coefficient of 60640 M^−1^cm^−1^. The final concentration of 3 µM rhodanese was used in experiments.

Thermal aggregation of substrate protein CS or rhodanese alone or in the presence of different concentrations rh Bri3 BRICHOS (determined with UV-Vis spectroscopy at 280 nm) was followed by measuring the change in the absorbance at 360 nm during 45 °C incubation for 2 h. Aggregation kinetics were measured in black, clear-bottom half-area polystyrene 96-well plates with non-binding surface (Corning Glass 3881, USA) in triplicates and using FLUOStar Galaxy plate reader (BMG Labtech, Offenberg, Germany). Samples were prepared in triplicates and all measurements were repeated 3 times. The same procedure was followed for the rh Bri2 BRICHOS. Rh Bri3 BRICHOS and rh Bri2 BRICHOS protein concentrations were also quantified by BCA protein assay (PierceTM, Thermo Scientific, USA) and Bradford based assay (Bio-Rad Protein Assay) following the manufacturer instructions. Different dilutions of rh BRICHOS (stock concentrations determined by UV-Vis spectrophotometric measurement) were compared (Supplementary Fig. S3). BCA and Bradford assay corrected BRICHOS protein concentrations were then plotted against the normalised final intensities in both non-fibrillar protein aggregation assays (Supplementary Fig. S4).

To study potential complex formation between rh Bri3 BRICHOS domain and CS after thermal aggregation, samples of CS:BRICHOS, 1:2 molar ratio, and also CS and rh Bri3 BRICHOS alone were collected after 2 h incubation at 45 °C, centrifuged for 5 min at 20,000 x g; supernatant and pellet fractions were analysed by SDS-PAGE under reducing conditions. Soluble fractions of incubated and comparable fresh samples were analysed by SEC and individual collected fractions were analysed by reducing SDS-PAGE.

### Aβ42 aggregation kinetics by thioflavin T assay and kinetic analysis

Aggregation kinetics under quiescent conditions were studied by recording the ThT fluorescence intensity as a function of time in a FLUOStar Galaxy plate reader (BMG Labtech, Germany). The fluorescence was measured in black, clear-bottom half-area polystyrene 384-well microplates with non-binding surface (Corning Lifesciences, USA), at 37 °C, using bottom optics with 440 nm excitation filter and 480 nm emission filter. For amyloid fibril formation kinetics of monomeric Aβ42 ThT analysis was carried out. Samples were prepared and quickly mixed on ice and pipetted into 384-well microplates in 4 replicates. Measurements were carried out in volume of 20 µl with each well containing 3 μM Aβ42 monomer, rh Bri3 BRICHOS or rh Bri2 BRICHOS at different concentrations relative to Aβ42, 10 μM ThT in 0.02% NaN_3_, 0.2 mM EDTA and 20 mM PB pH 8.0. Also, the aggregation kinetics of Aβ42 monomers with different concentrations from 1.5, 2.0, 2.5, 3.0, 4.0, 5.0, 7.0 to 9 µM in the presence of a constant concentration (3 µM) of rh Bri3 BRICHOS species were measured in the same manner.

First, the time evolution of fibril formation of 3 µM Aβ42 with different concentrations of Bri3 BRICHOS between 0 to 100% were fitted to a sigmoidal equation $F={F}_{0}+A/(1+\exp [{r}_{max}({\tau }_{1/2}-t)])$, from which half time ${t}_{1/2}$ and maximal growth rate *r*_*max*_ were extracted^31^. Second, the fibrillization kinetics was analysed by the nucleation model that is determined by primary (*k*_*n*_) and secondary nucleation (*k*_2_) events as well as for elongation (*k*_+_)^36^. Kinetic profiles at different Aβ42 monomer concentration were fitted globally, where the fitting parameters *λ* (for primary nucleation) and *κ* (for secondary nucleation) are dependent on nucleation rates by $\lambda =\sqrt{2\cdot {k}_{+}{k}_{n}\cdot m{(0)}^{{n}_{C}}}$ and $\kappa =\sqrt{2\cdot {k}_{+}{k}_{2}\cdot m{(0)}^{{n}_{2}+1}}$. The parameters *n*_*C*_ and *n*_2_ are the reaction orders for primary and secondary nucleation, respectively. In this model, the time dependence of the fibril mass *M(t)* is given by Eq. (1), where the additional coefficients are functions of *λ* and *κ*^36^. We also performed a global fit of the experimental data set at constant Aβ42 concentration and different Bri3 BRICHOS concentration, in which the fit was constrained such that one fitting parameter was held to a constant value across all Bri3 BRICHOS concentration, while the second parameter was the only free parameter. This fitting constraint results in a sole fitting rate constant *i.e. k*_*n*_, *k*_+_ or *k*_2_^31^.

1 $$\frac{M(t)}{M(\infty )}=1-{\left(\frac{{B}_{+}+{C}_{+}}{{B}_{+}+{C}_{+}\cdot \exp (\kappa t)}\cdot \frac{{B}_{-}+{C}_{+}\cdot \exp (\kappa t)}{{B}_{-}+{C}_{+}}\right)}^{\frac{{k}_{\infty }^{2}}{\kappa {\tilde{k}}_{\infty }}}\cdot \exp (-{k}_{\infty }t)$$

### Size-exclusion chromatography of Bri3 BRICHOS

Size-exclusion chromatography (SEC) was conducted using a Superose 6 10/300 GL column with ÄKTA pure 25 FPLC system (GE Healthcare Life Sciences, UK). Rh Bri3 BRICHOS samples were run at a flow rate of 0.5 ml·min^−1^ using 20 mM PB, 0.2 mM EDTA at pH 8.0 or 20 mM ammonium acetate buffer, pH 8.0 and the eluates were monitored at 280 nm. Calibration curves from LMW and HMW Gel Filtration Calibration Kits (GE Healthcare, UK) were used for the determination of the hydrodynamic radius. Bri3 hydrodynamic diameter was derived from the Stokes radius obtained by comparison to a standard curve derived from elution volumes of standard proteins with known Stokes radii.

For analyses of CS and rh Bri3 BRICHOS complex formation, 500 μL of each sample were eluted in 20 mM ammonium acetate buffer, pH 8.0 using flow rate of 0.7 ml·min^−1^.

## Supplementary information

Supplementary Information.

## Data Availability

The datasets generated during and/or analysed during the current study are available from the corresponding author on reasonable request.

## References

[1] Balchin D, Hayer-Hartl M, Hartl FU (2016). *In vivo* aspects of protein folding and quality control. Science.

[2] Hartl FU, Bracher A, Hayer-Hartl M (2011). Molecular chaperones in protein folding and proteostasis. Nature.

[3] Sunde M, Blake C (1997). The structure of amyloid fibrils by electron microscopy and X-ray diffraction. Adv. Protein Chem..

[4] Goldschmidt L, Teng PK, Riek R, Eisenberg D (2010). Identifying the amylome, proteins capable of forming amyloid-like fibrils. Proc Natl Acad Sci USA.

[5] Fändrich M, Fletcher MA, Dobson CM (2001). Amyloid fibrils from muscle myoglobin. Nature.

[6] Benson MD (2018). Amyloid nomenclature 2018: recommendations by the International Society of Amyloidosis (ISA) nomenclature committee. Amyloid.

[7] Hardy J, Selkoe DJ (2002). The amyloid hypothesis of Alzheimer’s disease: progress and problems on the road to therapeutics. Science.

[8] Ballard C (2011). Alzheimer’s disease. Lancet.

[9] Walsh DM (2002). Naturally secreted oligomers of amyloid beta protein potently inhibit hippocampal long-term potentiation *in vivo*. Nature.

[10] Haass C, Selkoe DJ (2007). Soluble protein oligomers in neurodegeneration: lessons from the Alzheimer’s amyloid beta-peptide. Nat Rev Mol Cell Biol.

[11] Sanchez-Pulido L, Devos D, Valencia A (2002). BRICHOS: a conserved domain in proteins associated with dementia, respiratory distress and cancer. Trends Biochem. Sci..

[12] Hedlund J, Johansson J, Persson B (2009). BRICHOS - a superfamily of multidomain proteins with diverse functions. BMC Res Notes.

[13] Johansson H, Nerelius C, Nordling K, Johansson J (2009). Preventing amyloid formation by catching unfolded transmembrane segments. J. Mol. Biol..

[14] Knight SD, Presto J, Linse S, Johansson J (2013). The BRICHOS domain, amyloid fibril formation, and their relationship. Biochemistry.

[15] Buxbaum JN, Johansson J (2017). Transthyretin and BRICHOS: The Paradox of Amyloidogenic Proteins with Anti-Amyloidogenic Activity for Abeta in the Central Nervous System. Front Neurosci.

[16] Nerelius C (2008). Mutations linked to interstitial lung disease can abrogate anti-amyloid function of prosurfactant protein C. Biochem. J..

[17] Vidal R (1999). A stop-codon mutation in the BRI gene associated with familial British dementia. Nature.

[18] Vidal R (2000). A decamer duplication in the 3’ region of the BRI gene originates an amyloid peptide that is associated with dementia in a Danish kindred. Proc Natl Acad Sci USA.

[19] Peng S, Fitzen M, Jornvall H, Johansson J (2010). The extracellular domain of Bri2 (ITM2B) binds the ABri peptide (1-23) and amyloid beta-peptide (Abeta1-40): Implications for Bri2 effects on processing of amyloid precursor protein and Abeta aggregation. Biochem. Biophys. Res. Commun..

[20] Willander H (2012). BRICHOS domains efficiently delay fibrillation of amyloid beta-peptide. J. Biol. Chem..

[21] Poska H (2016). Dementia-related Bri2 BRICHOS is a versatile molecular chaperone that efficiently inhibits Abeta42 toxicity in Drosophila. Biochem. J..

[22] Kurudenkandy FR (2014). Amyloid-beta-induced action potential desynchronization and degradation of hippocampal gamma oscillations is prevented by interference with peptide conformation change and aggregation. J. Neurosci..

[23] Cohen SI (2015). A molecular chaperone breaks the catalytic cycle that generates toxic Abeta oligomers. Nat Struct Mol Biol.

[24] Hermansson E (2014). The chaperone domain BRICHOS prevents CNS toxicity of amyloid-beta peptide in Drosophila melanogaster. Dis Model Mech.

[25] Nerelius C, Gustafsson M, Nordling K, Larsson A, Johansson J (2009). Anti-amyloid activity of the C-terminal domain of proSP-C against amyloid beta-peptide and medin. Biochemistry.

[26] Oskarsson ME (2018). BRICHOS domain of Bri2 inhibits islet amyloid polypeptide (IAPP) fibril formation and toxicity in human beta cells. Proc Natl Acad Sci USA.

[27] Vidal R (2001). Sequence, genomic structure and tissue expression of Human BRI3, a member of the BRI gene family. Gene.

[28] Martin L, Fluhrer R, Haass C (2009). Substrate requirements for SPPL2b-dependent regulated intramembrane proteolysis. J. Biol. Chem..

[29] Dolfe L (2018). The Bri2 and Bri3 BRICHOS Domains Interact Differently with Aβ42 and Alzheimer Amyloid Plaques. Journal of Alzheimer’s Disease Reports.

[30] Arosio P (2016). Kinetic analysis reveals the diversity of microscopic mechanisms through which molecular chaperones suppress amyloid formation. Nat Commun.

[31] Chen G (2017). Bri2 BRICHOS client specificity and chaperone activity are governed by assembly state. Nat Commun.

[32] Chen G (2020). Augmentation of Bri2 molecular chaperone activity against amyloid-β reduces neurotoxicity in mouse hippocampus *in vitro*. Communications Biology.

[33] Yang J (2015). The I-TASSER Suite: protein structure and function prediction. Nat Methods.

[34] Willander H (2012). High-resolution structure of a BRICHOS domain and its implications for anti-amyloid chaperone activity on lung surfactant protein C. Proc Natl Acad Sci USA.

[35] Hawe A, Sutter M, Jiskoot W (2008). Extrinsic fluorescent dyes as tools for protein characterization. Pharm. Res..

[36] Cohen SI (2013). Proliferation of amyloid-beta42 aggregates occurs through a secondary nucleation mechanism. Proc Natl Acad Sci USA.

[37] Matsuda S, Senda T (2019). BRI2 as an anti-Alzheimer gene. Med Mol Morphol.

[38] Wickham L, Benjannet S, Marcinkiewicz E, Chretien M, Seidah NG (2005). Beta-amyloid protein converting enzyme 1 and brain-specific type II membrane protein BRI3: binding partners processed by furin. J. Neurochem..

[39] Martin L (2008). Regulated intramembrane proteolysis of Bri2 (Itm2b) by ADAM10 and SPPL2a/SPPL2b. J. Biol. Chem..

[40] Matsuda S (2005). The familial dementia BRI2 gene binds the Alzheimer gene amyloid-beta precursor protein and inhibits amyloid-beta production. J. Biol. Chem..

[41] Matsuda S, Giliberto L, Matsuda Y, McGowan EM, D’Adamio L (2008). BRI2 inhibits amyloid beta-peptide precursor protein processing by interfering with the docking of secretases to the substrate. J. Neurosci..

[42] Matsuda S, Matsuda Y, D’Adamio L (2009). BRI3 inhibits amyloid precursor protein processing in a mechanistically distinct manner from its homologue dementia gene BRI2. J. Biol. Chem..

[43] Matsuda S, Matsuda Y, Snapp EL, D’Adamio L (2011). Maturation of BRI2 generates a specific inhibitor that reduces APP processing at the plasma membrane and in endocytic vesicles. Neurobiol. Aging.

[44] Tamayev R, Matsuda S, Arancio O, D’Adamio L (2012). beta- but not gamma-secretase proteolysis of APP causes synaptic and memory deficits in a mouse model of dementia. EMBO Mol Med.

[45] Biverstal H (2015). Dissociation of a BRICHOS trimer into monomers leads to increased inhibitory effect on Abeta42 fibril formation. Biochim. Biophys. Acta.

[46] Pettersen EF (2004). UCSF Chimera–a visualization system for exploratory research and analysis. J Comput Chem.

[47] Walsh DM (2009). A facile method for expression and purification of the Alzheimer’s disease-associated amyloid beta-peptide. FEBS J.

[48] Haslbeck M, Buchner J (2015). Assays to characterize molecular chaperone function *in vitro*. Methods Mol. Biol..

