# Supplementary material for: Recombinant Bri3 BRICHOS domain is a molecular chaperone with effect against amyloid formation and non-fibrillar protein aggregation
Source: Sci Rep. 2020 Jun 17;10:9817. doi: 10.1038/s41598-020-66718-y (PMC7299998; doi:10.1038/s41598-020-66718-y)
Supplement: Supplementary file 1 — Supplementary Information. [file 41598_2020_66718_MOESM1_ESM.pdf]

Supplementary Information

Recombinant Bri3 BRICHOS domain is a molecular chaperone with effect against amyloid formation and non-fibrillar protein aggregation

Helen Poska[1,#], Axel Leppert[2], Helene Tigro[1], Xueying Zhong[3], Margit Kaldmäe[1,4], Harriet E Nilsson[3,†], Hans Hebert[3], Gefei Chen[2], Jan Johansson[2,*]

[1]School of Natural Sciences and Health, Tallinn University, Tallinn, Estonia

[2]Department of Neurobiology, Care Sciences and Society, Division of Neurogeriatrics, Karolinska Institutet, Huddinge, Sweden

[3]School of Engineering Sciences in Chemistry, Biotechnology and Health, Department of Biomedical Engineering and Health Systems, KTH Royal Institute of Technology, Department of Biosciences and Nutrition, Karolinska Institutet, Huddinge, Sweden.

[4]Department of Microbiology, Tumor and Cell Biology, Karolinska Institutet Biomedicum, Solna, Sweden

[#]Current address: Department of Neurobiology, Care Sciences and Society, Division of Neurogeriatrics, Karolinska Institutet, Huddinge, Sweden

SUPPLEMENTARY FIGURES

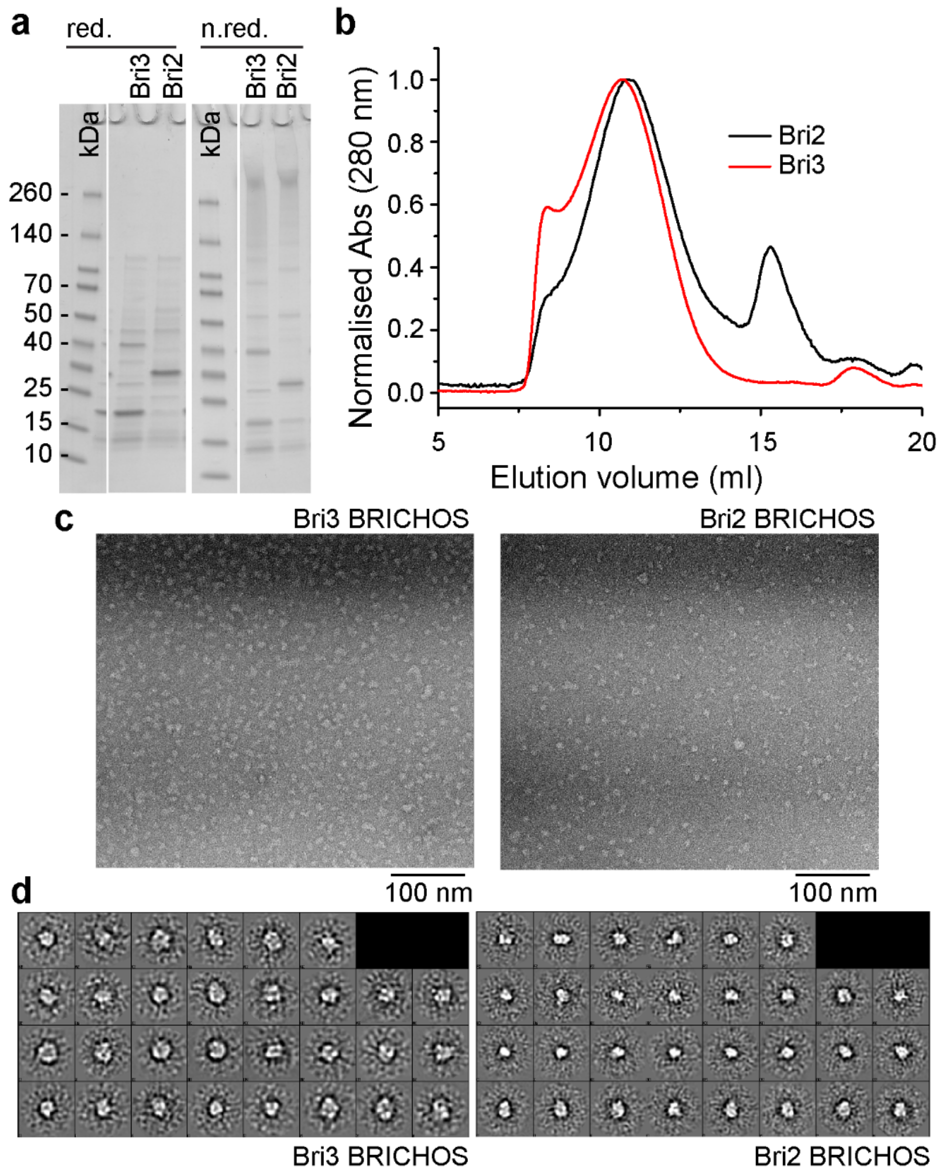

Supplementary Figure S1.

Size comparisons of rh Bri and Bri3 BRICHOS oligomers.

(a) SDS-PAGE of crude rh Bri3 and rh Bri2 BRICHOS under reducing and non-reducing conditions. Non-adjacent lanes of the same gel are separated by white vertical space, and un-cropped full-length gel is presented in Supplementary Figure S8d. **(b)** SEC of rh for rh Bri2 and rh Bri3 BRICHOS on Superpose 6 in ammonium acetate buffer. **(c)** Ttransmission electron micrographs of negatively stained rh Bri3 BRICHOS and rh Bri2 BRICHOS. **(d)** 2D classes of rh Bri3 and rh Bri2 BRICHOS oligomers.

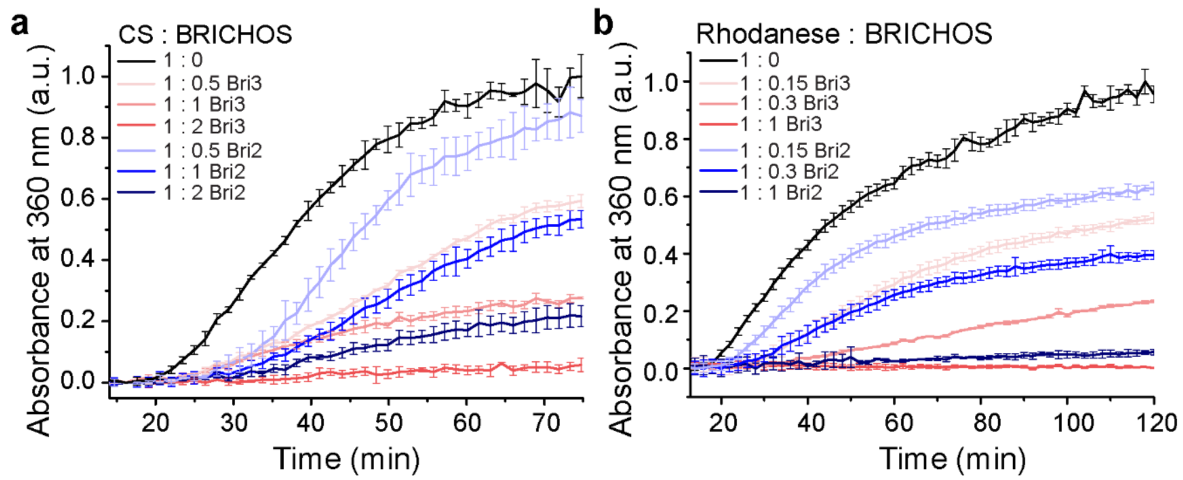

Supplementary Figure S2.

Thermally-induced non-fibrillar aggregation of model substrates is suppressed by rh Bri3 BRICHOS and rh Bri2 BRICHOS. (a) Aggregation of 600 nM citrate synthase (CS) alone (black) and in the presence of different molar ratios of rh Bri3 BRICHOS (shades of red) or rh Bri2 BRICHOS (shades of blue) at 45 ºC. **(b)** Aggregation of 3 µM rhodanese alone (black) or in the presence of different molar ratios of rh Bri3 BRICHOS (shades of red), Bri2 BRICHOS (shades of blue) at 45 ºC. Data are presented as mean ± SD of triplicates and the final intensities are used to derive the graphs in Fig. 3b and d.

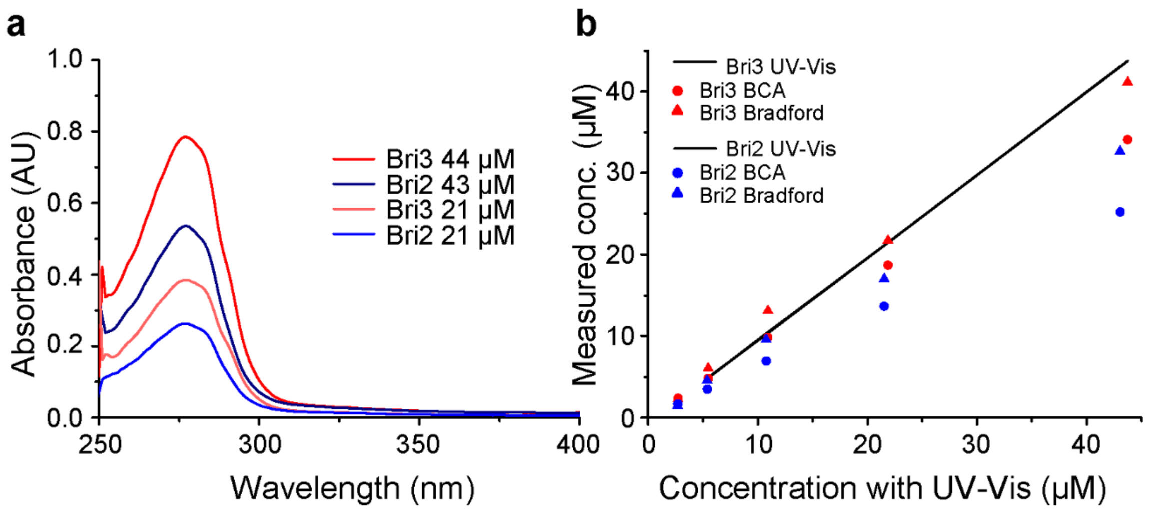

Supplementary Figure S3.

Absorbance and concentratiom determination of rh Bri2 and Bri3 BRICHOS. (a) UV-Vis absorption spectra of rh Bri3 BRICHOS (light and dark red) and rh Bri2 BRICHOS (light and dark blue). **(b)** Comparison of protein concentrations determined for rh BRICHOS samples using spectrophotometry (UV absorption at 280 nm), bicinchoninic acid (BCA) and Bradford assays.

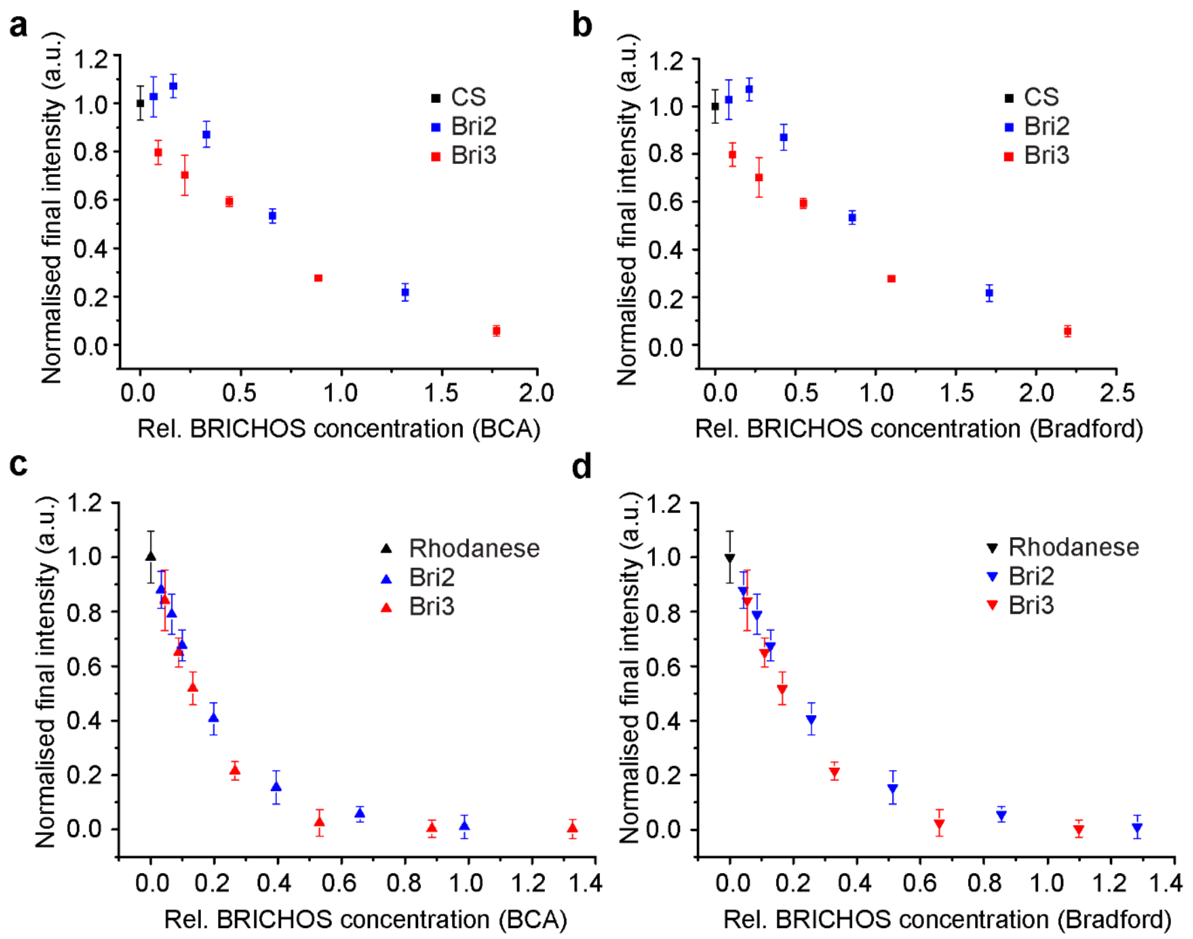

Supplementary Figure S4.

Rh Bri3 and Bri2 BRICHOS suppression of non-fibrillar protein aggregation BCA and Bradford derived concentrations. (a, b) The final intensity of CS aggregation alone (black) or in the presence of different molar ratios of rh Bri3 (red) or Bri2 BRICHOS (blue). **(c, d)** The final intensity of rhodanese aggregation alone (black) or in the presence of different molar ratios of rh Bri3 (red) and Bri2 BRICHOS (blue). BRICHOS concentrations are derived from BCA assay (**a, c**) or Bradford assay (**b, d**) shown in Supplementary Fig. S3b.

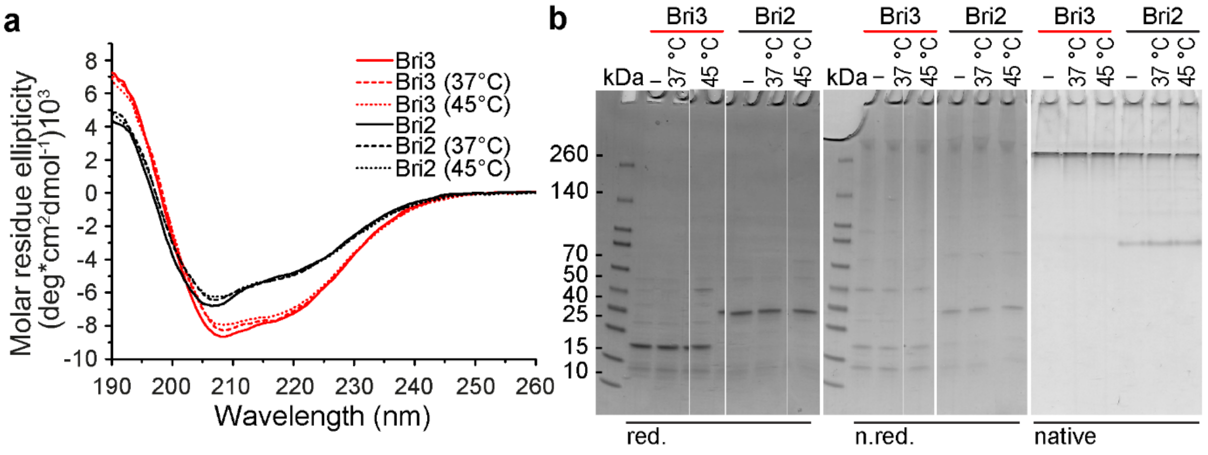

Supplementary Figure S5.

Rh Bri2 and Bri3 BRICHOS domain structures at different temperatures. (a) CD spectra of rh Bri3 BRICHOS (red) and rh Bri2 BRICHOS (black) measured at 25 °C, after incubation (dashed lines) for 24 h at 37 ºC or 2 h at 45 ºC (dotted lines). **(b)** Rh Bri3 and Bri2 BRICHOS before (-) and after incubation for 24 h or 37 ºC and 2 h at 45 ºC on reducing (red.), non-reducing (n.red.) SDS-PAGE and Native-PAGE. Non-adjacent lanes on gels are separated by white vertical space. Full size gels of native, reducing and non-reducing gels are presented in Supplementary Fig. S8e-f.

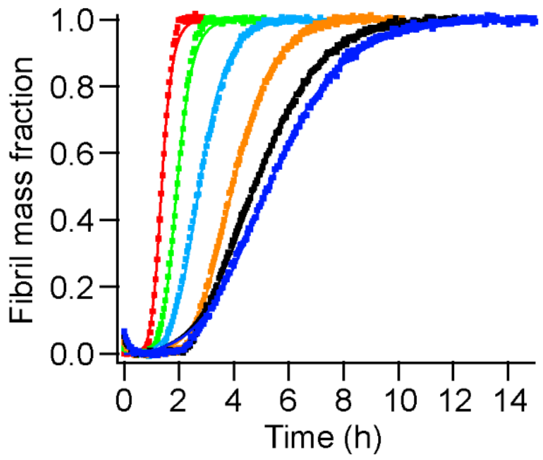

Supplementary Figure S6.

Rh Bri2 BRICHOS inhibition of Aβ42 fibril formation. Individual fits (solid lines) of normalized and averaged aggregation traces (dots) of 3 µM Aβ42 in the presence of 1:0 (red), 1:0.1 (green), 1:0.3 (cyan), 1:0.5 (orange), 1:0.7 (black) and 1:1 (blue) molar ratio of rh Bri2 BRICHOS with the combined rate constants $\sqrt{k_n k_+}$ and $\sqrt{k_+ k_2}$ as free fitting parameters.

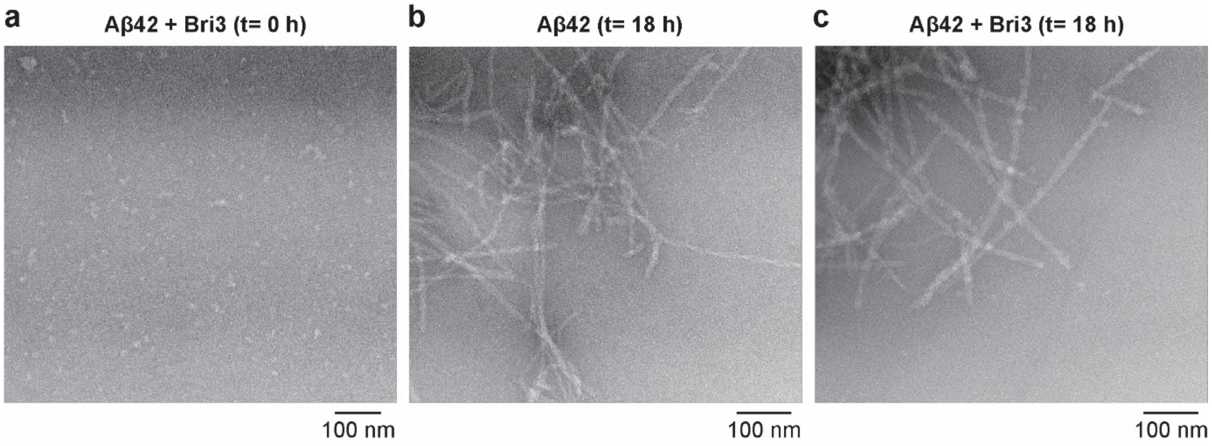

Supplementary Figure S7.

TEM micrographs of fibrillated Aβ42 in the absence and presence of rh Bri3 BRICHOS. 5 µM Aβ42 monomers mixed with rh Bri3 BRICHOS (1:0.7 Aβ42:BRICHOS molar ratio) at timepoint 0 h **(a)**, Aβ42 alone after 18 h **(b)** and Aβ42 with Bri3 BRICHOS incubated for 18 h **(c)**.

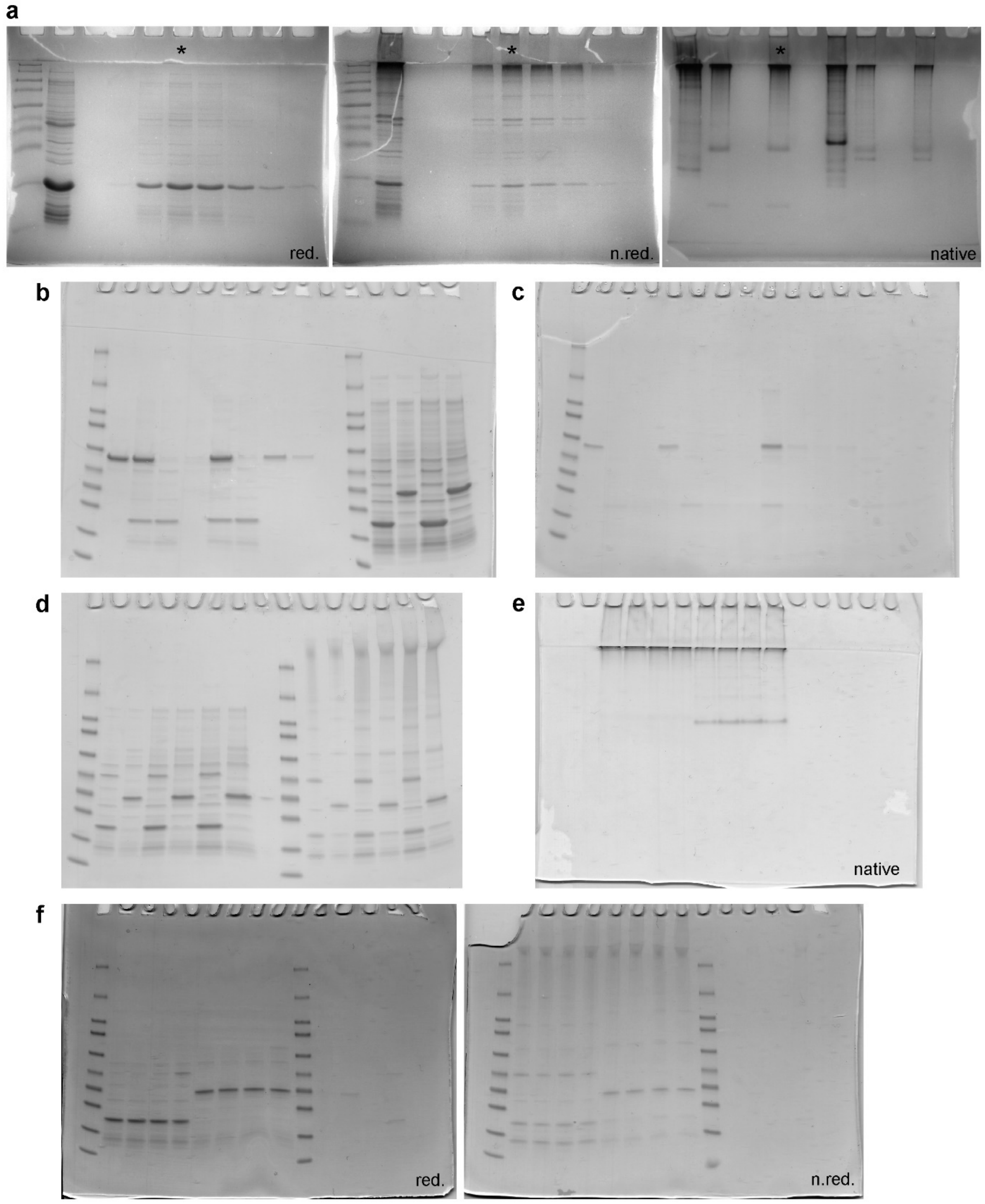

Supplementary Figure S8.

Full size representations of gels. (a) reducing (red.), non-reducing (n.red.) SDS-PAGE and native PAGE of rh Bri3 BRICHOS oligomers shown in Fig. 2b. Lanes marked with * are presented in the main figure. Reducing SDS-PAGE gels shown in **(b)** Fig. 4a and **(c)** Fig. 4c, representing Bri3 BRICHOS complex formation with partly denatured CS. **(d)** Crude rh Bri3

and rh Bri2 BRICHOS under reducing and non-reducing conditions shown in Supplementary Fig. 1a. **(e)** native-PAGE and **(f)** reducing (red.), non-reducing (n.red.) SDS-PAGE gels of rh Bri3 and Bri2 BRICHOS before and after incubation presented first in Supplementary Fig. 5b.
